# A novel *PARD3B-NUTM1* fusion in an aggressive primary CNS embryonal tumor in a young adult

**DOI:** 10.1186/s40478-020-00991-w

**Published:** 2020-07-17

**Authors:** Kyungmin Ko, Takashi Kitani, Brent T. Harris, Amjad N. Anaizi, David Solomon, Arie Perry, Jeffrey Toretsky, Metin Ozdemirli

**Affiliations:** 1 Department of Pathology and Laboratory Medicine, Georgetown University Hospital, 3900 Reservoir Rd NW, Washington, DC 20007 USA; 2 Departments of Oncology and Pediatrics, Georgetown University School of Medicine, 3970 Reservoir Rd NW, Washington, DC 20057 USA; 3 Department of Neurology, MedStar Georgetown University Hospital, 3800 Reservoir Rd NW, Washington, DC 20007 USA; 4 Department of Neurosurgery, MedStar Georgetown University Hospital, 3800 Reservoir Rd NW, Washington, DC 20007 USA; 5 Department of Pathology, University of California, San Francisco, 505 Parnassus Ave, San Francisco, CA 94143 USA

**Keywords:** CNS embryonal tumor, NUT carcinoma, *NUTM1*, Next-generation sequencing

## Main text

**Fig. 1:**
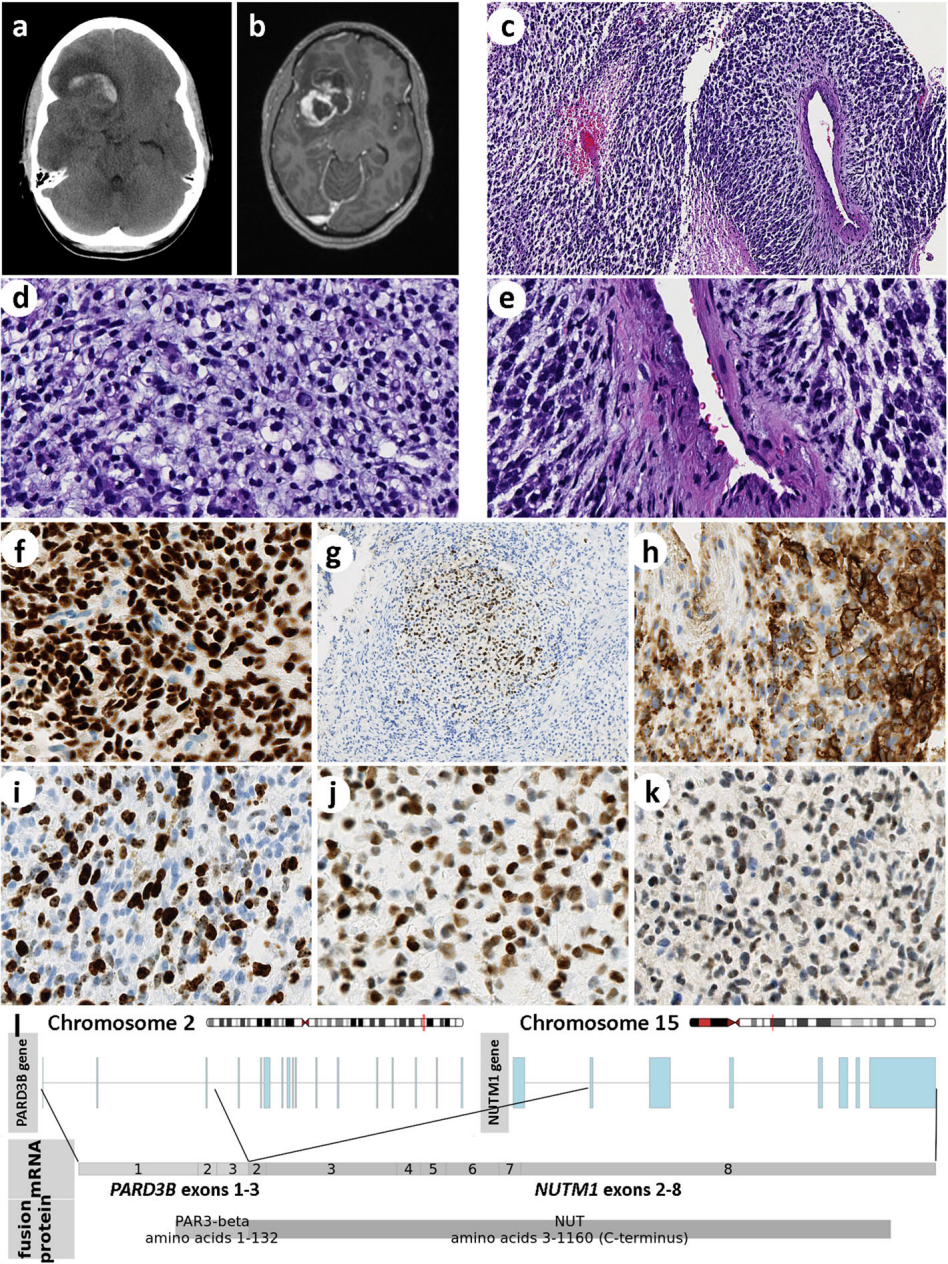
CT and MRI showed a frontotemporal mass with hemorrhage and surrounding vasogenic edema (a, b). Representative histopathology. Low power view shows variegated appearance (c). Small, primitive-appearing tumor cells with some cytoplasmic clearing and hyperchromatic round-to-oval nuclei (d). Spindle cells with ovoid nuclei and myxoid background condensed around a vessel (e). Diffuse uniform nuclear NUT immunostaining (f). A focus of GFAP expression (g). CD99 stains all tumor cells in a membranous or cytoplasmic dot-like pattern (h). Ki-67 proliferative index is high (i). There is nuclear p53 (j) and c-Myc (k) expression in most tumor cells. PARD3B-NUTM1 gene fusion, discovered by NGS of tumor DNA, predicts a fusion protein (l)

We report a novel *PARD3B-NUTM1* gene fusion in a primary embryonal tumor of the brain which had a very aggressive course. A 29-year-old female presented with worsening headache of three-weeks duration. CT and MRI showed a hemorrhagic mass involving the right inferior frontal lobe and temporal lobe (Fig. 1 a, b). The tumor was well-demarcated. It was surgically removed with no gross residual tumor. MRI on post-operative day 27 showed recurrence and additional tumor in the prepontine region and between cerebellar tonsils. Chemotherapy with vincristine, cisplatin, and cyclophosphamide was initiated and staged re-excisions were performed, but the patient expired due to intraventricular hemorrhage on post-operative day 34. A comprehensive postmortem examination revealed no residual viable tumor in the brain or any other extracranial tumor.

Microscopic sections showed a relatively well-circumscribed, moderately cellular neoplasm. The tumor had a variegated histology with foci showing primitive, spindle cells in a myxoid to fibrillar background, foci showing small epithelioid cells with clear cytoplasm around thin-walled vessels, and foci of microvascular proliferation and necrosis (Fig. 1 c-e). There was microscopic infiltration of adjacent brain parenchyma by single tumor cells and reactive gliosis. Mitoses were frequent. No well-differentiated islands of squamous epithelium characteristic of NUT midline carcinoma were observed. By immunohistochemistry (Figs. 1 f-k), there was diffuse CD56 expression, patchy dot-like and strong membranous expression of CD99, focal neurofilament expression and GFAP was positive in a small subset of epithelioid tumor cells. Synaptophysin was positive in single scattered cells (less than 1%). Chromogranin A, OLIG2, IDH1 R132H mutant protein, EMA, pan-keratin, p40, p63, CD34, progesterone receptor, HMB-45, Melan-A, SOX10, desmin, smooth muscle actin, muscle specific actin, CD10, L1CAM, and WT-1 were negative by immunohistochemistry. INI-1 and ATRX expression were retained. The tumor cells showed strong nuclear p53 and c-Myc expression in most tumor cells and had a high Ki-67 (MIB-1) proliferative index (approximated at 60%). We classified the tumor as “central nervous system embryonal tumor, not otherwise specified”. The tumor was negative for *EWSR1* rearrangement by fluorescence in situ hybridization (FISH). Histology of post-chemotherapy re-excision specimens were similar to the original with focal minimal necrosis (in approximately 10% of the tumor).

Next-generation sequencing (NGS) using the UCSF500 panel was performed (as described in reference [1]), which revealed a novel *PARD3B-NUTM1* gene fusion between *PARD3B* intron 3–4 and *NUTM1* intron 1–2 resulting in an in-frame fusion of exons 1–3 of *PARD3B* and exons 2–8 of *NUTM1*. The sequence predicts a fusion protein comprising the N-terminal 132 amino acids of the partitioning defective 3 homolog B protein (PAR3-β) and almost the entire NUT protein (amino acids 3–1160 C -terminus) (Fig. 1 l). There was also a *BRCA2* nonsense mutation (p.K944*, c.1830A > T), which was present at a heterozygous allele frequency. NGS also showed chromosomal copy number changes that included gains of 6p and interstitial 15q, and loss of 6q. However, NGS did not detect TP53 or MYC alterations including mutation, amplification or rearrangements despite p53 and c-myc protein overexpression. Subsequent immunohistochemistry showed diffuse strong nuclear expression of NUT protein.

## Discussion

NUT carcinoma is an aggressive midline carcinoma predominantly seen in the head and neck region with undifferentiated or poorly differentiated morphology characterized by NUT expression [2]. The prognosis of NUT carcinomas is poor, with a median survival of 9.8 months [3]. It is now apparent that *NUTM1* gene fusions also characterize a subset of undifferentiated soft tissue and visceral tumors not restricted to carcinomas or to the midline [4] and also has been described in B-ALL [5] . *NUTM1*-rearranged tumors often exhibit rapid growth and spread. Histologically they are heterogeneous and sometimes variegated and have a poorly differentiated cytology. In the brain, several *NUTM1* rearrangements have been reported (Table 1). Five of the six cases reported as examples of a newly defined entity called “CNS Ewing sarcoma family tumor with *CIC* alteration” were shown to express NUT protein by immunohistochemistry and two of these were shown to have *CIC-NUTM1* fusion [6]. An exceptional case of a *NUTM1-*rearranged brain tumor resulted in a disease-free survival at 16 months [7]. Our case showed a very aggressive course in the setting of a novel *PARD3B-NUTM1* fusion accompanied by chromosomal copy number changes. PAR3-beta, the product of *PARD3B*, regulates cell-cell contact and indirectly activates the Hippo pathway, but few studies implicate *PARD3B* in tumorigenesis [8, 9].

**Table 1:** Comparison of reported NUTM1-rearranged primary brain tumors

	Fusion gene	Age Sex Location	Histology	IHC profile	Course	Discovery	Reference
Cases 1 & 2 *	*CIC-NUTM1* *(2 cases)*	Not available	Small-cell phenotype, alveolar and fascicular growth	NUT (strong).	Not available	RNA sequencing of select cases	6
Case 3	*BRD4-NUTM1*	3, Male, Parietal lobe	Small round cells. Epithelioid-polygonal cells with a reticular-alveolar pattern and prominent myxoid stroma. Nuclear molding, speckled chromatin and conspicuous mitotic activity.	GFAP (2+, focal), synaptophysin (1+), NUT (5+). **Negative**: pan-keratin, HMWK, LMWK, C4, p63, chromogranin	Died of disease 12 months post-op with chemotherapy	Retrospective RNA sequencing of undifferentiated tumors with nuclear isomorphism	4
Case 4	*ATXN1-NUTM1*	21, Female, Frontal lobe	Fascicular architecture and chondro-myxoid areas; some neuron-like tumor cells; large nucleoli	NUT, GFAP (strong), p53, CD56, **Negative**: OLIG2, S100, TTF1, chromogranin, synaptophysin, CD34, p63, CK5/6, SMA. **Wild type**: ATRX, INI1, BRG1	Disease -free 16 months post-op	RNA sequencing of a brain tumor after classification by methylation profile and NUT IHC.	7
Case 5	*PARD3B-NUTM1*	29, Female, Frontal lobe	Variegated tumor consisting mostly of small epithelioid cells with myxoid or fibrillar background	NUT, CD99, CD56, p53, GFAP (focal), neurofilament (focal). **Negative**: Keratin, p63, desmin, S-100, chromogranin A, synaptophysin (only rare cells positive), OLIG2, IDH R132H, EMA, SOX10, actins, **Wild-type**: INI-1, ATRX	Died of disease one month post-op	DNA sequencing panel	Current case

*Features of cases 1 and 2 are based on overall description of CNS Ewing sarcoma family tumor with *CIC* alteration cases reported in reference [6]

The clinical and histopathologic features of the *NUTM1*-rearranged brain tumors reported to date are not specific and pose a diagnostic challenge. For the three cases with available histologic description, the tumors showed young age at presentation and hyperchromatic nuclei [4, 7]. Cytologic features were dissimilar. A myxoid background component was a common feature. GFAP expression varied from focal to diffuse. The reported cases were consistently negative for cytokeratin and chromogranin A, and at most focally positive for synaptophysin. There was strong nuclear expression of NUT protein expressed in all five cases. In contrast, some soft tissue and visceral *NUTM1*-rearranged tumors were negative for NUT expression by immunohistochemistry. The reported NUTM1-rearranged brain tumors were not designated as a specific entity. Based on the morphologic features, intra-axial location, lack of other primary site, diffuse CD99 and CD56 and focal GFAP and neurofilament positivity, lack of carcinoma and sarcoma markers, and lack of histologic and molecular alterations that define other specific CNS primitive neuroectodermal tumors, our case was classified as “CNS embryonal tumor, not otherwise classified” category in the current WHO classification system, which is equivalent to CNS primitive neuroectodermal tumor in previous WHO classification systems.

The reported cases of *NUTM1*-rearrangements in primary brain tumors were discovered by NGS (Table 1). RNA sequencing was performed either retrospectively or for research purposes for cases 1, 2, and 3. RNA sequencing was performed for diagnostic workup in case 4 (*ATXN1-NUTM1)*. In the current case, DNA sequencing was performed for diagnostic workup, revealing additional chromosomal copy number changes.

To our best knowledge, *PARD3B-NUTM1* fusion has not been reported in any tumor. As our case illustrates, utilization of NGS for routine diagnostic workup of primitive appearing CNS tumors may uncover more *NUTM1*-rearranged tumors with different fusion partners. Molecular analysis of these aggressive tumors may explain the differences seen in histopathologies and provide clues to prognosis and identification of novel therapies.

## Data Availability

Not applicable.

## Abbreviations

FISH: Fluorescence in-situ hybridization
NGS: Next generation sequencing
